# Acupuncture for somatosensory deficits after stroke: a systematic review and meta-analysis

**DOI:** 10.3389/fmed.2025.1504215

**Published:** 2025-02-07

**Authors:** Shumeng Ren, Yuhang Chen, Yu Liu, Qiuyi Lv, Jing Peng, Lei Song, Yihuai Zou, Hua Zhang, Xing Chen

**Affiliations:** ^1^Dongzhimen Hospital, The First Affiliated Hospital of Beijing University of Chinese Medicine, Beijing, China; ^2^Department of Neurology and Stroke Center, Dongzhimen Hospital, The First Affiliated Hospital of Beijing University of Chinese Medicine, Beijing, China

**Keywords:** acupuncture, stroke, somatosensory deficits, systematic review, meta-analysis

## Abstract

**Objective:**

This meta-analysis aimed to evaluate the randomized controlled trials (RCTs) of acupuncture for somatosensory deficits after stroke to provide the current best evidence for clinical practice.

**Methods:**

A systematic search was performed for eligible articles on the acupuncture for somatosensory deficits after stroke based on 14 databases. Two reviewers independently screened the RCTs, extracted data, and evaluated the methodological quality. A meta-analysis of RCTs was conducted using STATA 11.0 software.

**Results:**

A total of 57 RCTs were included. Meta-analysis results showed that compared with the control group, the acupuncture group had a higher effective rate [risk ratio (RR) = 1.21, 95% CI (1.17, 1.24), *I*^2^ = 10.6%, *P* < 0.001] and a better sensory disturbance score [mean difference (MD) = 5.37, 95%CI (3.81, 6.92), *I*^2^ = 93.9%, *P* < 0.001]. The Visual Analog Scale in the acupuncture group was lower [MD = −1.44, 95%CI (−1.81, −1.07), *I*^2^ = 94.5%, *P* < 0.001]. The acupuncture also showed an improvement in the ability of daily living [MD = 12.19, 95% CI (8.50, 15.87), *I*^2^ = 97.2%, *P* < 0.001] and the symptoms of neurological deficit [Standardized Mean Difference (SMD) = −1.53, 95%CI (−2.04, −1.03), *I*^2^ = 89.7%, *P* < 0.001].

**Conclusions:**

The current best evidence shows that acupuncture has advantages in raising the effective rate, alleviating sensory disturbance, relieving pain, enhancing the ability of daily living, and improving the symptoms of neurological deficits for somatosensory deficits after stroke compared with the control group. And the adverse reactions in acupuncture group were acceptable. However, further well-designed high-quality and multicenter international studies are needed to verify the effectiveness and safety of acupuncture for somatosensory deficits after stroke.

**Systematic review registration:**

https://www.crd.york.ac.uk/prospero/display_record.php?ID=CRD42020186040.

## Introduction

Stroke, a collective term for a category of diseases caused by cerebrovascular disorders leading to neurological dysfunction, mainly includes ischemic stroke and hemorrhagic stroke (1). It is one of the major diseases causing death and disability in humans, bringing a heavy burden to many patients, families, and society. The incidence of stroke in China continues to rise, with new cases each year accounting for a quarter of the global total. Research from the Global Burden of Disease (GBD) (2) shows that in 2019, there were 3.94 million new stroke cases in our country, with a total of 28.76 million stroke patients and 2.19 million stroke-related deaths.

Following the onset of apoplexy, the five principal symptoms are sudden fainting, hemiplegia, deviation of the mouth and tongue, speech impairment or aphasia, and numbness on one side of the body (3). Sensory disorders are common sequelae subsequent to brain meridian damage, with approximately 50% to 80% of stroke patients presenting varying degrees of sensory disorders (4). The course is frequently protracted and difficult to be cured, severely influencing the recovery of patients' motor functions and other neurological functions, as well as the quality of life. The findings from Lv et al.'s (5) study have demonstrated that the parietal cortex and certain subcortical regions, including the insular cortex and thalamus, contribute to somatosensory symptoms. Current rehabilitation approaches mainly revolve around modern rehabilitation technologies and equipment, which are costly and lack precise individualized rehabilitation plans. Simultaneously, rehabilitation treatment relies on professionals and the environment, and its accessibility is not high (6). Acupuncture, a traditional Chinese medicine technique, was incorporated into the applicable scope of stroke rehabilitation by the World Health Organization in 1998. The possible neural mechanism underlying the modulation of acupuncture is primarily located within the somatosensory cortex (7), in connection with structural neuroplasticity in the primary somatosensory cortex (8) and modulating the neuronal death pathway (9). Additionally, the efficacy and safety of acupuncture for stroke have been a subject of ongoing controversy. Kong et al.'s (10) systematic review did not reveal a positive effect of acupuncture as a treatment for functional recovery after stroke. Park et al.'s (11) systematic review showed that the most methodologically rigorous trials of these included nine studies demonstrated no significant effect of acupuncture. In contrast, Zhang et al.'s (12) overview indicated that acupuncture might be effective in treating post-stroke neurological impairments and dysfunctions, such as dysphagia, but it cannot assist in preventing post-stroke death or disability, nor can it improve other aspects of stroke recovery, such as post-stroke motor dysfunction. In the benchmarks for the practice of acupuncture set by the WHO (13), it is stated that there has been a deficiency of standardized protocols for acupuncture treatment by which the practice can be compared and evaluated. As a result, the efficacy and safety of acupuncture for somatosensory deficits after stroke have remained inconclusive thus far. This study aims to conduct a meta-analysis of published randomized controlled trials (RCTs) on acupuncture treatment for sensory disorders after stroke, to explore the effects of acupuncture intervention on sensory disorders after stroke, and to provide more scientific and reliable evidence for clinical application.

## Materials and methods

### Registration

The protocol for this systematic review and meta-analysis was registered in PROSPERO (No. CRD42020186040) and followed the Preferred Reporting Items for Systematic Reviews and Meta-Analyses (PRISMA) guidelines.

### Literature search

Two researchers (YHC and YL) independently searched PubMed, Cochrane Library, Embase, Web of Science, ClinicalTrials.gov, CINAHL, Wiley, OVID, PROQUES, SCOPUS, Chinese National Knowledge Infrastructure (CNKI), Chinese Science and Technique Journals Database (VIP), Wanfang Database, and SinoMed from study inception to December 1, 2023. There was no restriction on language. The search terms were “stroke,” “somatosensory deficits,” “acupuncture,” “randomized controlled trial,” and related terms. Details of the search strategies are shown in Table 1.

**Table 1:** Search strategies in PubMed.

**#**	**Term**
#1	Stroke OR Strokes OR Cerebrovascular Accident OR Cerebrovascular Accidents OR CVA (Cerebrovascular Accident) OR CVAs (Cerebrovascular Accident) OR Cerebrovascular Apoplexy OR Apoplexy, Cerebrovascular OR Vascular Accident, Brain OR Brain Vascular Accident OR Brain Vascular Accidents OR Vascular Accidents, Brain OR Cerebrovascular Stroke OR Cerebrovascular Strokes OR Stroke, Cerebrovascular OR Strokes, Cerebrovascular OR Apoplexy OR Cerebral Stroke OR Cerebral Strokes OR Stroke, Cerebral OR Strokes, Cerebral OR Stroke, Acute OR Acute Stroke OR Acute Strokes OR Strokes, Acute OR Cerebrovascular Accident, Acute OR Acute Cerebrovascular Accident OR Acute Cerebrovascular Accidents OR Cerebrovascular Accidents, Acute
#2	Cerebral Hemorrhage OR Hemorrhage, Cerebrum OR Cerebrum Hemorrhage OR Cerebrum Hemorrhages OR Hemorrhages, Cerebrum OR Cerebral Parenchymal Hemorrhage OR Cerebral Parenchymal Hemorrhages OR Hemorrhage, Cerebral Parenchymal OR Hemorrhages, Cerebral Parenchymal OR Parenchymal Hemorrhage, Cerebral OR Parenchymal Hemorrhages, Cerebral OR Intracerebral Hemorrhage OR Hemorrhage, Intracerebral OR Hemorrhages, Intracerebral OR Intracerebral Hemorrhages OR Hemorrhage, Cerebral OR Cerebral Hemorrhages OR Hemorrhages, Cerebral OR Brain Hemorrhage, Cerebral OR Brain Hemorrhages, Cerebral OR Cerebral Brain Hemorrhage OR Cerebral Brain Hemorrhages OR Hemorrhage, Cerebral Brain OR Hemorrhages, Cerebral Brain
#3	Brain Ischemia OR Brain Ischemias OR Ischemia, Brain OR Ischemic Encephalopathy OR Encephalopathy, Ischemic OR Ischemic Encephalopathies OR Cerebral Ischemia OR Cerebral Ischemias OR Ischemias, Cerebral OR Ischemia, Cerebral
#4	Cerebral Infarction OR Cerebral Infarctions OR Infarctions, Cerebral OR Infarction, Cerebral OR Cerebral Infarct OR Cerebral Infarcts OR Infarct, Cerebral OR Infarcts, Cerebral OR Cerebral Infarction, Hemisphere OR Hemisphere, Infarction, Cerebral OR Infarction, Hemisphere, Cerebral OR Hemisphere, Cerebral Infarction OR Cerebral, Hemisphere, Infarction OR Infarction, Cerebral, Hemisphere OR Subcortical Infarction OR Infarction, Subcortical OR Infarctions, Subcortical OR Subcortical Infarctions OR Posterior Choroidal Artery Infarction OR Anterior Choroidal Artery Infarction OR Cerebral Infarction, Right Hemisphere OR Right Hemisphere, Cerebral Infarction OR Infarction, Right Hemisphere, Cerebral OR Right Hemisphere, Infarction, Cerebral OR Cerebral, Right Hemisphere, Infarction OR Infarction, Cerebral, Right Hemisphere
#5	Cerebrovascular Disorders OR Cerebrovascular Disorder OR Vascular Diseases, Intracranial OR Intracranial Vascular Disease OR Intracranial Vascular Diseases OR Vascular Disease, Intracranial OR Intracranial Vascular Disorders OR Intracranial Vascular Disorder OR Vascular Disorder, Intracranial OR Vascular Disorders, Intracranial OR Cerebrovascular Diseases OR Cerebrovascular Disease OR Disease, Cerebrovascular OR Diseases, Cerebrovascular OR Brain Vascular Disorders OR Brain Vascular Disorder OR Vascular Disorder, Brain OR Vascular Disorders, Brain OR Cerebrovascular Occlusion OR Cerebrovascular Occlusions OR Occlusion, Cerebrovascular OR Occlusions, Cerebrovascular OR Cerebrovascular Insufficiency OR Cerebrovascular Insufficiencies OR Insufficiencies, Cerebrovascular OR Insufficiency, Cerebrovascular
#6	#1 OR #2 OR #3 OR #4 OR #5
#7	Somatosensory Disorders OR Somatosensory Disorder OR Somatic Sensation Disorders OR Sensation Disorder, Somatic OR Sensation Disorders, Somatic OR Somatic Sensation Disorder OR Pain Sensation Diminished OR Diminished, Pain Sensation OR Diminisheds, Pain Sensation OR Pain Sensation Diminisheds OR Sensation Diminished, Pain OR Sensation Diminisheds, Pain OR Thermal Sensation Disorders OR Sensation Disorder, Thermal OR Sensation Disorders, Thermal OR Thermal Sensation Disorder OR Position Sense Disorders OR Position Sense Disorder OR Sense Disorder, Position OR Sense Disorders, Position OR Proprioceptive Disorders OR Proprioceptive Disorder OR Impairment, Light Touch Sensation OR Light Touch Sensation Impairment OR Pinprick Sensation Diminished OR Diminished, Pinprick Sensation OR Diminisheds, Pinprick Sensation OR Pinprick Sensation Diminisheds OR Sensation Diminished, Pinprick OR Sensation Diminisheds, Pinprick
#8	Sensation Disorders OR Sensation Disorder OR Special Senses Disorders OR Senses Disorder, Special OR Senses Disorders, Special OR Special Senses Disorder OR Sensory Disorders OR Sensory Disorder
#9	#7 OR #8
#10	Acupuncture OR Acupuncture Therapy OR Acupuncture Treatment OR Acupuncture Treatments OR Treatment, Acupuncture OR Therapy, Acupuncture OR Pharmacoacupuncture Treatment OR Treatment, Pharmacoacupuncture OR Pharmacoacupuncture Therapy OR Therapy, Pharmacoacupuncture OR Acupotomy OR Acupotomies
#11	randomized controlled trial OR controlled clinical trial OR clinical trials as topic OR random allocation OR double-blind method OR single-blind method OR clinical trial OR research design OR comparative study OR evaluation studies OR follow-up studies OR prospective studies OR cross-over studies OR clinical trial
#12	#6 AND #9 AND #10 AND #11

### Inclusion criteria

(1) Study types

RCTs of acupuncture for somatosensory deficits after stroke without restriction in language.

(2) Participants

Participants, without gender, age, region, or course restrictions, were diagnosed with stroke by domestic or international relevant diagnostic standards.

(3) Interventions

Acupuncture alone or combined with comparisons. Acupuncture treatments are specified as needle-based acupuncture, including but not limited to manual, electro-acupuncture, fire acupuncture, warm acupuncture, ear (auricular) acupuncture, head acupuncture, and more.

(4) Comparisons

Conventional pharmacotherapy, other non-pharmacotherapy, or invalid groups, including placebo and no treatment.

(5) Outcomes

The included studies reported at least one primary outcome: effective rate and sensory disturbance score. The secondary outcomes included Visual Analog Scale (VAS), daily living ability score, neurological deficit severity score, and incidence of adverse events.

### Exclusion criteria

Studies with incomplete data or duplicate publications.

### Data extraction

Two researchers (QYL and JP) independently screened the titles, abstracts, and full texts of the retrieved studies for eligibility and independently extracted the data of the final included literature. Disagreements were resolved by mutual negotiation or by consultation with a third researcher (LS). The following information was extracted: authors, publication year, general information, participants' characteristics, details of interventions (type of acupuncture, acupoints, frequency, duration of treatment, retention time of acupuncture), and outcomes.

### Risk of bias assessment

Two researchers (SMR and XC) independently assessed the methodological quality of the included studies using the Cochrane Risk of Bias Tool 2.0 (RoB 2.0) (14), which contains six aspects: randomization, deviations from the intended interventions, missing outcome data, measurement of the outcome, selective outcome reporting, and overall bias. Each aspect was evaluated as “low risk of bias” “some concerns” or “high risk of bias.” Disagreements were resolved by mutual negotiation or by consultation with other researchers (LS). The appraisal of acupuncture procedures was assessed by Revised Standards for Reporting Interventions in Clinical Trials of Acupuncture (STRICTA).

### Assessing certainty of the evidence

The Grading of Recommendations Assessment, Development and Evaluation (GRADE) system (15) was used to rank the quality of evidence in five downgrading domains: risk of bias, inconsistency, indirectness, imprecision, and publication bias. The quality of the evidence was classified into four grades: high, moderate, low, or very low.

### Statistical analysis

All statistical analyses were performed with STATA Software, version 11.0. We used a random-effects model (DerSimonian and Laird method) to calculate summary effect estimates for primary and secondary outcomes. Heterogeneity was assessed by the χ^2^ test and the *I*^2^ statistic. Dichotomous outcomes were expressed as the risk ratio (RR) with a 95% confidence interval (CI), while continuous outcomes were expressed as the standardized mean difference (MD) with a 95% CI. Standardized mean difference (SMD) was selected when different measures and units were used, or when the difference in means between studies was too large. A funnel plot and Egger's test were applied to evaluate publication bias when the number of included studies was more than 10. A subgroup analysis and a meta-regression were conducted based on the interventions, comparisons, type of disease, course of disease and course of treatment. Sensitivity analysis was conducted to evaluated the stability of the results.

## Results

### Included studies

**Figure 1:**
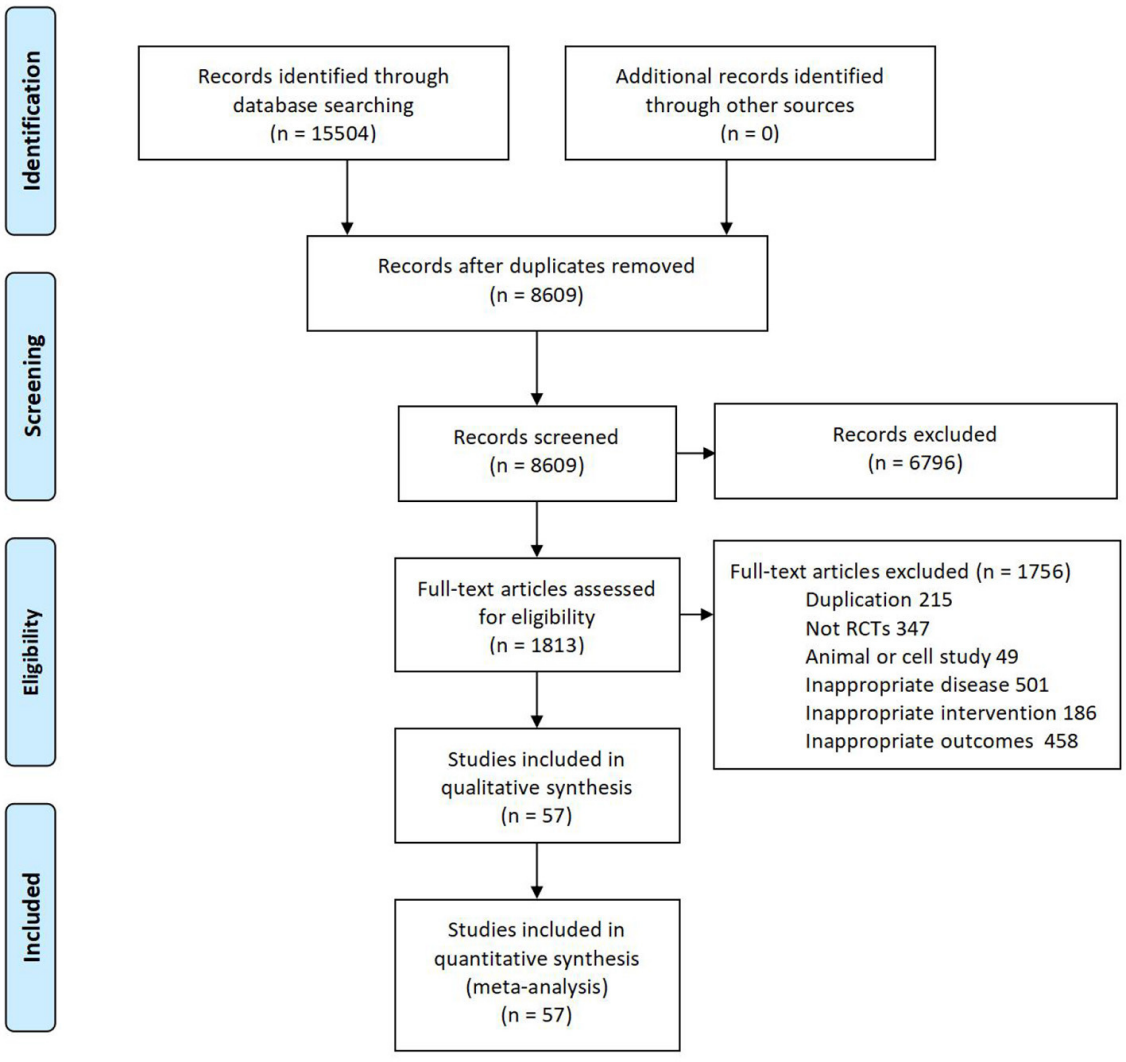
Flow diagram of included studies.

Fifteen thousand five-hundred four articles were yielded by database searching. Eventually, 57 eligible RCTs (with data for 4,794) were included after screening full texts according to the inclusion and exclusion criteria. And a flow diagram for study selection is shown in Figure 1. The 57 articles were all published between 1999 and 2023. Among these articles, 24 articles reported foundation and were all funded by industry. For details of each included trial please see the Table 2.

**Table 2:** Characteristics of included studies.

**Study ID**	**Sample size (male/female) Mean age (years)**		**Type of disease**	**Course of disease**	**Interventions**		**Frequency**		**Course of treatment (days)**	**Outcomes**
	**T**	**C**			**T**	**C**	**T**	**C**		
LvSS 2021	24/16 58.85 ± 5.36	23/17 58.79 ± 5.28	CD	Convalescence	WA+rehabilitation	Rehabilitation	1 session 2 days	1 session daily	28	
LiuSW 2020	22/19 55.27 ± 3.63	21/20 55.16 ± 3.74	ICD	Sequelae	EA+BT	BT	1 session daily	1 session daily	15	
YuYY 2020	20/15 60.07 ± 5.44	19/16 60.15 ± 5.58	CD	Convalescence	MA+BT	BT	1 session 2 days	NR	20	
JiangH 2020	16/14 58.25 ± 9.16	18/12 57.36 ± 9.20	ICD	Convalescence	MA+BT	BT	NR	NR	NR	
MaHM 2022	7/4 66.45 ± 8.98	6/5 64.55 ± 6.89	CD	Convalescence	FN+rehabilitation	Rehabilitation	1 session 2 days	1 session daily	14	
JiaZK 2021	16/14 68.50 ± 17.00	18/12 65.30 ± 17.00	CD	Convalescence	MA	Drug	NR	1 session daily	28	
HanWF 2022	29/21 41–75	29/21 41–75	ICD	Convalescence	MA	BT	1 session daily	NR	28	
ZhuLL 2020	19/11 60.83 ± 9.36	22/8 62.53 ± 8.87	CD	Convalescence	EA+rehabilitation	Rehabilitation	NR	NR	20	
XuL 2020	16/12 59.32 ± 10.68	11/17 55.00 ± 10.45	CD	Convalescence	MA+rehabilitation	Rehabilitation	1 session daily	1 session daily	56	
LiuY 2023	26/13 61.46 ± 4.96	24/15 59.50 ± 4.85	CD	Convalescence	EA+rehabilitation	Rehabilitation	1 session 2 days	5 sessions a week	28	
YanK 2020	23/19 67.56 ± 8.69	24/18 68.81 ± 9.17	ICD	Convalescence	MA+drug	Drug	1 session 2 days	3 sessions daily	28	
ZhengWF 2023	59/41 53.50 ± 3.15	58/42 53.00 ± 3.12	CD	Convalescence	MA+drug	Drug	1 session daily	2 sessions daily	56	
LiangMT 2023	12/18 55.20 ± 4.46	14/16 56.43 ± 5.53	CD	Convalescence	MA	Drug	1 session daily	2 sessions daily	28	
TanLM 2023	20/14 59.34 ± 10.01	18/16 59.73 ± 9.54	CD	Convalescence	MA	Drug	6 sessions a week	3 sessions daily	28	
YeZ 2021	11/9 57.20 ± 4.30	12/8 56.30 ± 4.60	CD	Convalescence	MA+rehabilitation	Rehabilitation	1 session daily	1 session daily	30	
ShiYH 2021	18/12 53.30 ± 7.60	16/14 54.20 ± 5.40	ICD	Convalescence	MA	BT	1 session daily	1 session daily	28	
WuSQ 2021	118/102 57.40 ± 5.20	119/101 57.40 ± 5.20	CD	Acute	MA+rehabilitation	Rehabilitation	1 session daily	1 session daily	56	
LiangYG 2021	16/14 65.07 ± 4.65	15/15 63.43 ± 5.83	ICD	Convalescence	MA	BT	1 session daily	1 session daily	28	
XieSS 2021	58/52 60.11 ± 5.19	57/51 59.61 ± 5.26	ICD	Acute	MA+BT	BT	1 session daily	1 session daily	28	
YangXH 2022	16/14 60.75 ± 4.26	17/13 60.82 ± 4.15	CD	Convalescence	MA+drug	Drug	1 session daily	1 session daily	30	
LiuCX 2023	22/19 58.19 ± 11.07	24/17 57.61 ± 10.23	ICD	Convalescence	MA+rehabilitation	BT	1 session daily	1 session daily	14	
WangC 2022 (53)	17/16 59.00 ± 7.00	19/15 60.00 ± 7.00	CD	Convalescence	MA+drug	Drug	1 session daily	2 sessions daily	28	
LuY 2021 (28)	31/25 59.16 ± 9.25	30/26 57.81 ± 8.73	ICD	Convalescence	MA+drug	Drug	1 session daily	3 sessions daily	28	
ZhangQX 2021	27/21 62.71 ± 10.14	28/20 61.28 ± 9.56	ICD	Convalescence	MA+drug	Drug	1 session daily	3 sessions daily	28	
ChaoY 2021	19/14 60.58 ± 8.09	21/13 60.32 ± 7.32	ICD	Convalescence	MA	Drug	1 session daily	NR	28	
WangWL 2022	16/16 56.26 ± 7.32	17/15 55.34 ± 7.51	CD	Convalescence	MA+BT	BT	2 sessions a week	1 session daily	28	
FangMF 2018	28/12 63.42 ± 9.12	25/15 62.54 ± 9.04	CD	Convalescence	MA+rehabilitation	Rehabilitation	1 session daily	1 session daily	24	
FuB 2019	24/18 52.31 ± 7.64	22/20 51.89 ± 7.85	ICD	Convalescence	MA+drug	Drug	1 session daily	3 sessions daily	20	
GuXD 2013	8/12 56.50 ± 7.10	9/11 54.90 ± 8.20	CD	Acute	EA+rehabilitation	Rehabilitation	1 session daily	2 sessions daily	36	
HouXY 2017	27/19 58.19 ± 7.38	26/20 57.47 ± 7.56	CD	Convalescence	MA+BT	BT	1 session daily	1 session daily	30	
JiangZY 1999	20/10 NR	21/9 NR	CD	Convalescence	EA	Drug	1 session daily	3 sessions daily	30	
KeJ 2015	23/17 51.60 ± 12.50	25/15 52.10 ± 10.60	CD	Acute	MA+rehabilitation	Rehabilitation	1 session daily	NR	28	
KongY 2018	18/3 64.30 ± 12.31	13/8 63.42 ± 11.26	CD	Convalescence	MA	Drug	2 sessions daily	2 sessions daily	28	
LanLK 2006	10/8 50–72	11/7 51–73	ICD	Acute	MA+BT	BT	5 sessions a week	NR	21	
LiuYF 2015	17/13 57.90 ± 9.90	19/11 56.10 ± 12.00	CD	Sequelae	MA+BT	BT	1 session daily	1 session daily	28	
LuM 2018	32/8 55.25 ± 4.39	30/10 59.43 ± 5.82	CD	Convalescence	EA	Drug	1 session daily	NR	28	
QiaoHZ 2019a	24/16 70.09 ± 6.87	23/17 70.41 ± 6.98	ICD	Convalescence	MA+drug	Drug	2 sessions daily	NR	56	
QiaoHZ 2019b	25/19 57.11 ± 8.16	26/18 56.90 ± 8.71	ICD	Acute	MA+BT	BT	1 session daily	1 session daily	28	
QiaoHZ 2018	34/21 63.50 ± 5.20	33/22 62.80 ± 4.90	CD	Convalescence	MA	BT	5 sessions a week	NR	28	
ShangYP 2019	17/13 57.23 ± 6.17	16/14 57.29 ± 6.12	CD	Convalescence	MA+rehabilitation	Rehabilitation	1 session daily	1 session daily	56	
ShiGB 2015	21/14 61.2	22/13 62.5	ICD	Convalescence	MA+BT	BT	1 session daily	NR	21	
ShiYJ 2018 (18)	NR NR	NR NR	CD	Convalescence	MA+rehabilitation	Rehabilitation	1 session daily	1 session daily	NR	
WangH 2015	14/18 68.69 ± 1.43	15/18 67.81 ± 1.47	CD	Sequelae	MA	Drug	5 sessions a week	3 sessions daily	28	
WangHB 2015	32/20 56.70 ± 2.50	29/23 54.20 ± 3.60	CD	Convalescence	MA+rehabilitation	Rehabilitation	1 session daily	NR	14	
WangSM 2018	14/16 55.40 ± 6.60	15/15 57.30 ± 5.80	CD	Convalescence	EA	Drug	1 session daily	2 sessions daily	60	
WangSP 2019	24/15 64.86 ± 10.88	22/17 64.88 ± 10.85	CD	Acute	MA+rehabilitation	Rehabilitation	1 session daily	NR	20	
WangWQ 2009	14/16 62.50 ± 4.40	14/15 63.10 ± 4.10	CD	Convalescence	MA	Drug	1 session daily	3 sessions daily	28	
WangX 2019	30/32 60.45 ± 8.13	32/29 59.69 ± 8.09	CD	Acute	MA+BT	BT	2 sessions daily	NR	28	
WangXM 2004	9/7 58.60 ± 5.10	10/6 59.10 ± 5.20	ICD	Convalescence	MA+BT	BT	5 sessions a week	NR	21	
WuXL 2001	31/19 64.16 ± 9.91	35/17 67.82 ± 10.64	CD	Convalescence	MA+BT	BT	1 session daily	NR	28	
XingYL 2007a	21/15 58.81 ± 7.51	22/14 61.50 ± 8.05	ICD	Acute	MA+rehabilitation	Rehabilitation	1 session daily	NR	56	
XingYL 2007b	20/16 59.44 ± 6.31	22/14 62.26 ± 7.82	ICD	Convalescence	MA	BT	1 session daily	NR	56	
ZhangX 2010	7/4 NR	7/4 NR	CD	Convalescence	MA	Drug	1 session daily	NR	10	
ZhangXR 2012	19/9 61.8	20/8 63.6	CD	Convalescence	EA	Drug	1 session daily	NR	30	
ZhangYE 2015	15/17 61.00 ± 6.00	17/15 61.00 ± 8.00	ICD	Convalescence	MA+BT	BT	NR	NR	56	
ZhengZT 2010	10/8 62.20 ± 8.70	10/8 66.01 ± 9.60	ICD	Convalescence	EA+rehabilitation	Rehabilitation	NR	NR	21	
ZhuJ 2019	21/19 68.82 ± 5.61	20/20 70.82 ± 4.71	CD	Acute	MA	BT	1 session daily	1 session daily	7	
ZhuQX 2014	38/25 30–70	36/21 32–68	CD	Convalescence	MA	Drug	1 session 2 days	NR	40	

T, treatment group; C, control group; NR, not reported; CD, cerebrovascular disease; ICD, ischemic cerebrovascular disease; WA, warm acupuncture; EA, electroacupuncture; BT, basic treatment, including brain protection, neurotrophic support, improvement of circulation, antiplatelet aggregation, lipid regulation and plaque stabilization, and maintenance of water, electrolyte, and acid-base balance; MA, manual acupuncture; FN, fire needle; rehabilitation, the combination of physiotherapy, psychotherapy, occupational therapy, speech and language therapy, cognitive rehabilitation, recreational therapy and nutritional counseling.

effective rate; sensory disturbance score; visual analog scale; daily living ability score; neurological deficit severity score; safety assessment; syndrome integral of traditional Chinese medicine; quality of life; hemorheological index; somatosensory evoked potential.

#### Participants

All included studies were conducted in China, and 55 studies were published in Chinese and two in English (16, 17). The sample sizes of the 57 trials ranged from 22 to 440. Three trials (16, 18, 19) did not report the mean age of participants, and the age of the rest of the participants ranged from 30 to 79 years. Forty-five trials included more males than females, ranging from 37% to 86% male. One trial (18) did not describe the gender of the participants. Twenty-one trials included only participants with ischemic stroke (20–39). All other trials included participants with ischemic and hemorrhagic strokes (16–18, 40–66). None of the included trials had a definition of severity. There were 10 trials (26, 32, 34, 37, 51, 56, 58, 67–69) involving participants with an interval from stroke onset < 2 weeks (acute phase), 44 trials (21–25, 27–31, 40–50, 52–55, 66) between 2 weeks and 6 months (convalescent phase), and three trials (17, 20, 63) including participants with an interval from stroke onset of more than 6 months (sequelae phase).

#### Interventions

In 38 studies, acupuncture therapy combined with the same intervention applied in the control group (20, 21, 23, 40–42, 44–47, 50, 51, 66) was compared with the control group; in the remaining 19 trials, acupuncture alone was compared with the control group's treatment (16, 22, 24, 25, 30, 43, 48, 49, 59–61, 63, 65, 66, 70). Among the included trials, there were 3 three-armed trials (37, 38, 59). In this review, the basic treatment included smoking and alcohol cessation, oxygen inhalation, as well as medication for regulating blood glucose, blood pressure, blood lipids, anticoagulation, or antiplatelet aggregation. The drugs included Methylcobalamin, Pregabalin, Amitriptyline, Carbamazepine and Gabapentin. The acupuncture interventions used varied considerably across trials. Forty-seven trials used manual stimulation (21–23, 41–43, 45, 47–50, 66), nine used electroacupuncture (16, 20, 39, 44, 46, 56, 60, 65, 71), and each had a trial using the fire needle (42) and the warm acupuncture (40). Acupuncture point prescriptions were not consistent, with 40 trials only involving body acupoints (20–22, 40–44, 47–50, 66), seven trials involving scalp acupoints (37, 38, 45, 56, 59, 62, 68), five trials involving wrist ankle acupoints (23, 31, 46, 55, 69), one trial involving eye acupoints (24), three trials using both body and scalp acupoints (52, 57, 65), one trial using body and wrist ankle acupoints (30) and one trial using body, scalp and wrist ankle acupoints (33). Through statistical analysis, it has been found that our study mentions a total of 43 acupoints. We discovered that baihui (DU20) appears the most frequently, with a total of 14 occurrences, followed by the sanyinjiao (SP6) with 13 occurrences, and then the zusanli (ST36) with nine occurrences. Fengchi (GB20), hegu (LI4), and yanglingquan (GB34) each appeared six times. Neiguan (PC6), quchi (LI11), and Ah Shi points each appeared five times. Jianyu (LI15), weizhong (BL40), jiquan (HT1), and xuehai (SP10) each appeared four times. Fenglong (ST40), tianzhu (BL10), and shenting (GV24) each appeared three times. Wangu (GB12), taichong (LR3), shousanli (LI10), waiguan (SJ5), huantiao (GB29), fengshi (GB31), chize (LU5), yongquan (KI1), liangqiu (ST34), dicang (ST4) and taiyang each appeared two times. The acupoints that appeared only once include yintang (GV29), taixi (KI3), pishu (BL20), xinshu (BL15), feishu (BL13), shenshu (BL23), ganshu (BL18), yuyao, jianzhen (SI9), xiaguan (ST7), jiache (ST6), chengjiang (ST24), sibai (ST2), yingxiang (LI20), qubin (GB7), and baxie. The needle retention time was 15 min to 8 h in all the included trials. The length of treatment period ranged from 7 to 60 days, with the number of treatment sessions varying from seven to 112 sessions and the frequency of treatment varying from two sessions per week to two sessions per day. The intervention details showed in Appendix 1.

#### Outcomes

The most commonly reported outcomes were effective rate, visual analog scale and daily living ability score. Forty-three trials evaluated the effective rate of acupuncture. The efficacy criteria for 20 trials were self-defined, and the remaining trials' were derived from the “Guiding Principles for Clinical Research of New Traditional Chinese Medicines,” “Diagnostic Criteria and Therapeutic Effect Standards for Syndromes and Diseases in Traditional Chinese Medicine,” “Criteria for Diagnosis, Cure, and Improvement of Clinical Diseases,” and “Guidelines for Rehabilitation Treatment of Stroke in China” and so on. Twenty-two trials evaluated the effect of acupuncture on activities of daily living. The measures employed included the Barthel Index (BI) or modified Barthel Index (MBI) and the Activities of Daily Living Scale (ADL). Fifteen trials reported sensory disturbance score. Nine trials measured the neurological deficit severity score. The measures employed included the National Institutes of Health Stroke Scale (NIHSS) and the Chinese stroke patient neurological deficit score, also known as the Modified Edinburgh-Scandinavian Stroke Scale (MESSS). Only eleven trials reported information on adverse events. None of the 57 included trials provided any information on death, the proportion of participants requiring institutional care, or extensive family support after acupuncture treatment or at the end of follow-up.

#### Risk of bias in included studies

**Figure 2:**
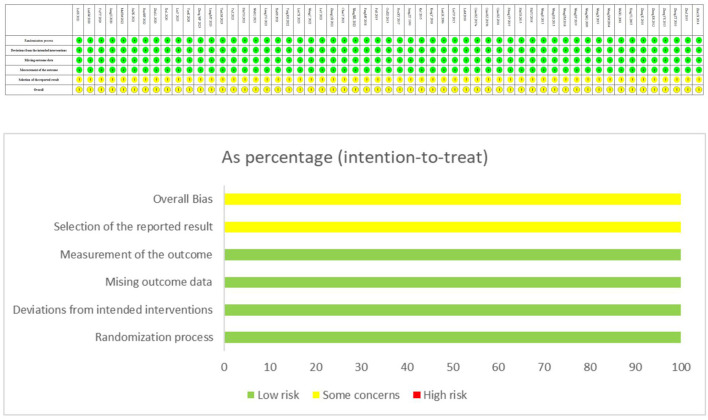
Risk of bias graph and summary.

The ROB assessment is shown in Figure 2.

(1) Allocation

(1.1) Random sequence generation

Thirty-five trials randomly assigned participants to groups by using random number tables. One trial generated random numbers through SPSS software and one assigned based on the order of consultation. The remaining 20 trials did not report their methods of random sequence generation.

(1.2) Allocation concealment

Of the 57 included trials, only one trial reported adequate allocation concealment by using sealed envelopes (45).

(2) Blinding

Due to the nature of acupuncture manipulations, blinding to participants is not suitable for acupuncture therapy. This makes all included studies have a certain risk of bias. Only one study specified data collectors and outcome assessors were masked to treatment allocation (72).

(3) Incomplete outcome data

Six trials reported withdrawals, but the results were not analyzed on an intention-to-treat basis. None of the remaining 51 trials made any mention of dropouts or withdrawals. For all 51 trials, the count of participants who were randomized matched the count of participants who were subsequently analyzed, indicating that no exclusions took place post-randomization.

(4) Selective reporting

The included trials in this review did not report some clinically important outcomes, such as death, requiring Institutional care, and all-cause mortality. Of the 57 included studies, only 11 studies reported adverse events, therefore we assumed that this may have constituted some degree of reporting bias.

(5) Other potential sources of bias

There was insufficient information reported to determine if there were any other potential sources of bias.

### Meta-analysis

#### Effective rate

**Figure 3:**
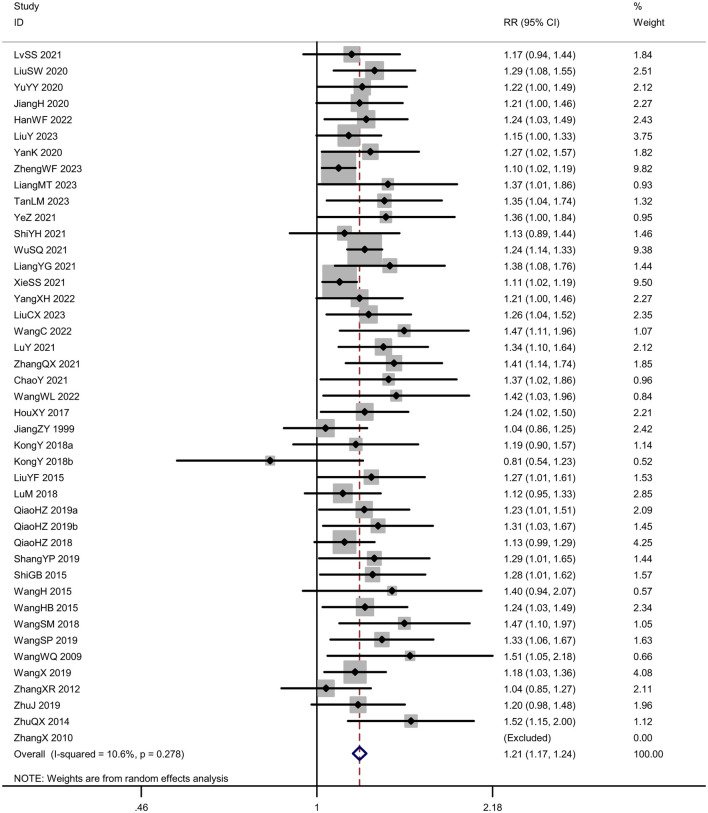
Forest plot of the effective rate.

**Figure 4:**
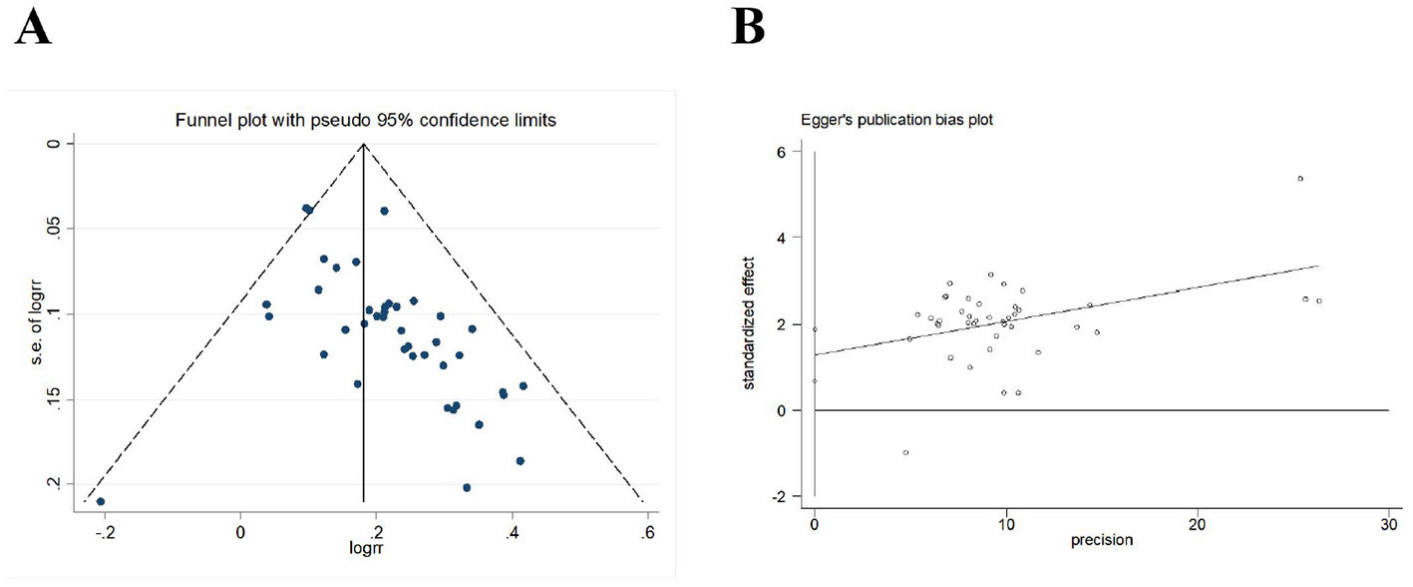
Publication bias in the effective rate. **(A)** Funnel plots. **(B)** Egger's test.

Forty-three trials (*N* = 3,798 participants) evaluated the effective rate of acupuncture for post-stroke somatosensory deficits. The results of meta-analysis indicated that acupuncture group was significantly higher than the control group [*RR* = 1.21, 95% *CI* (1.17, 1.24), *I*^2^ = 10.6%, *P* < 0.001] in improving effective rate (Figure 3). In this analysis there was no significant publication bias on Egger's test (*P* < 0.001; Figure 4).

#### Sensory disturbance score

**Figure 5:**
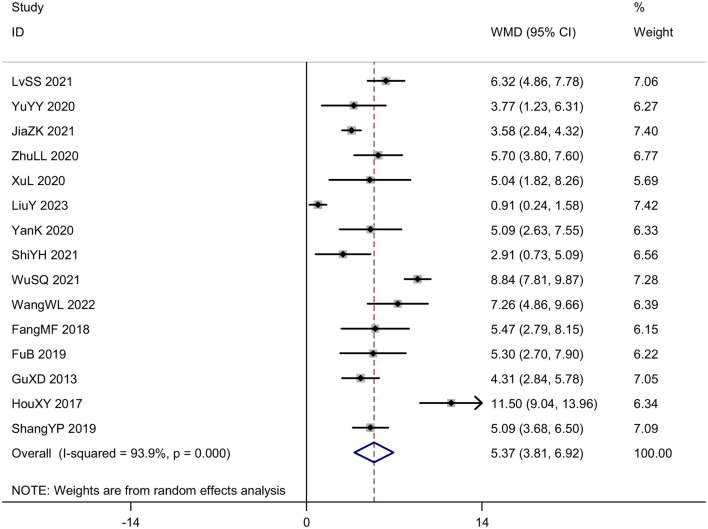
Forest plot of the sensory disturbance score.

**Figure 6:**
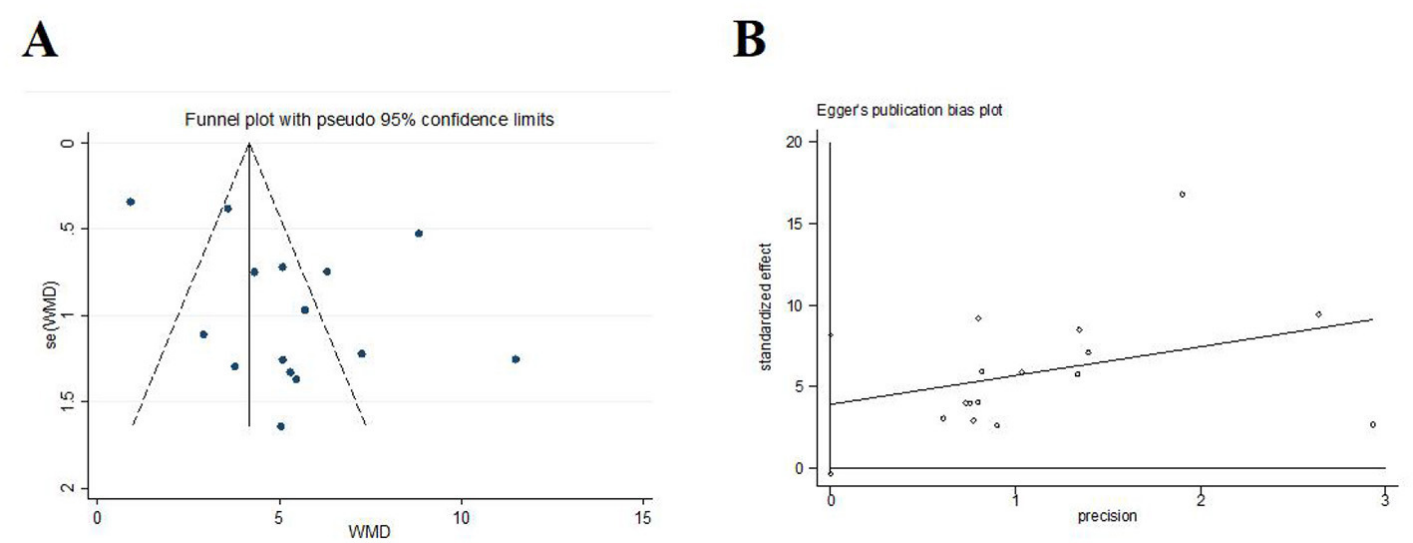
Publication bias in the sensory disturbance score. **(A)** Funnel plots. **(B)** Egger's test.

Fifteen trials (*N* = 1,407) assessed the severity of somatosensory deficits in stroke patients by using the sensory disturbance score. The meta-analysis results showed that acupuncture led to a better sensory disturbance score [*MD* = 5.37, 95% *CI* (3.81, 6.92), *I*^2^ = 93.9%, *P* < 0.001] than control group (Figure 5). Funnel plots and Egger's test were done to evaluate publication bias, and results showed no significant publication bias (*P* = 0.068; Figure 6).

#### Visual analog scale

**Figure 7:**
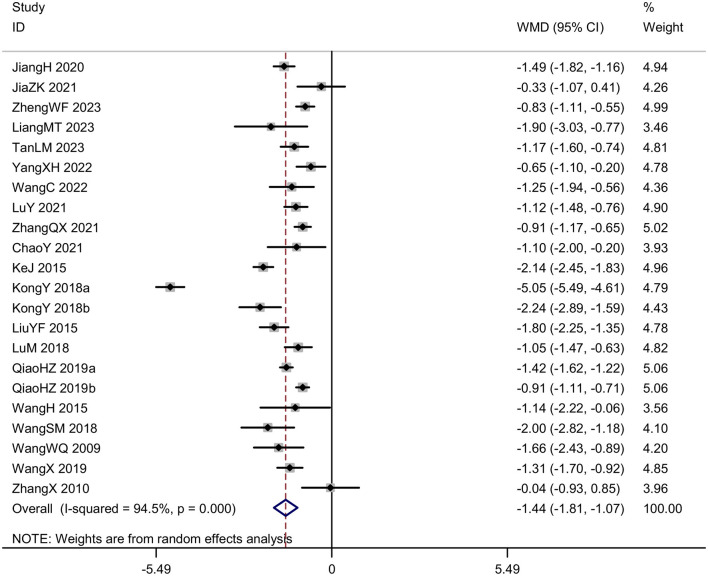
Forest plot of the visual analog scale.

**Figure 8:**
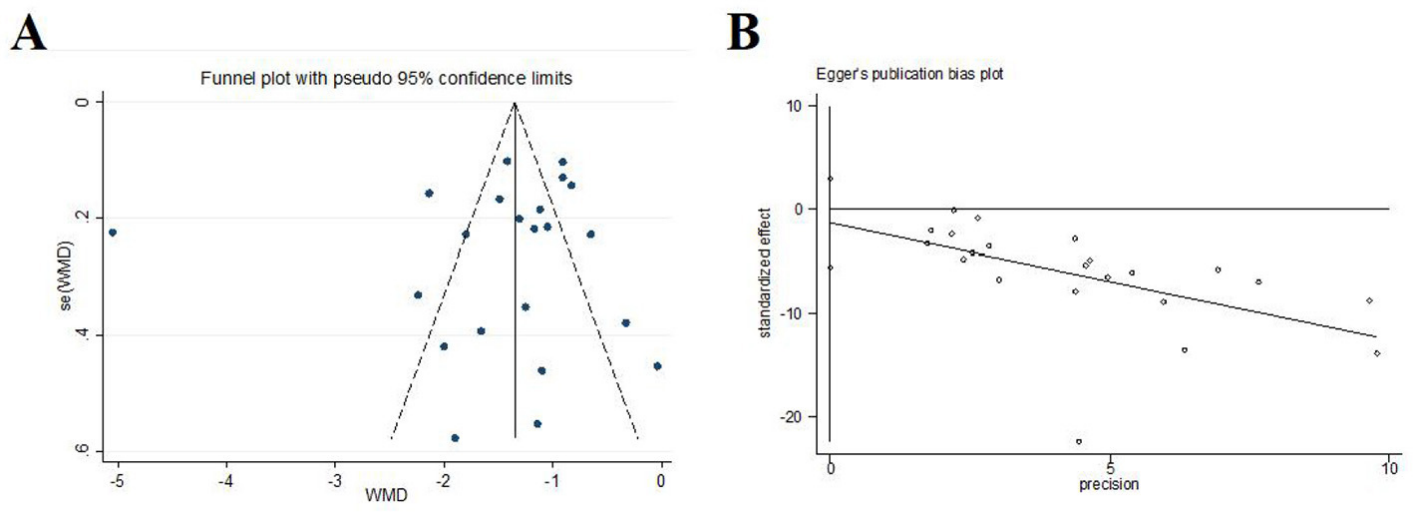
Publication bias in the visual analog scale. **(A)** Funnel plots. **(B)** Egger's test.

We conducted a meta-analysis of the study results based on the visual analog scale. In 22 trials (*N* = 1,651), acupuncture was statistically superior to control [*MD* = −*1.44, 95%CI* (–1.81, –1.07), *I*^2^ = 94.5%, *P* < 0.001; Figure 7]. Funnel plots and Egger's test were done to evaluate publication bias, and results showed no significant publication bias (*P* = 0.526; Figure 8).

#### Daily living ability score

**Figure 9:**
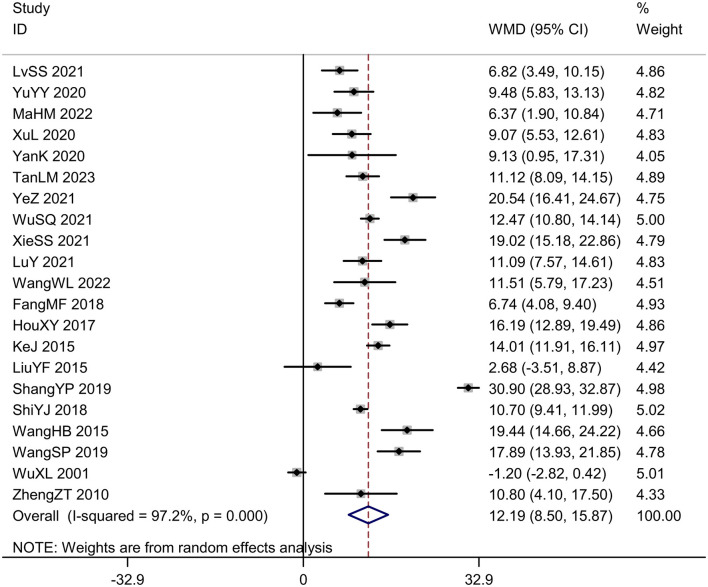
Forest plot of the daily living ability score.

**Figure 10:**
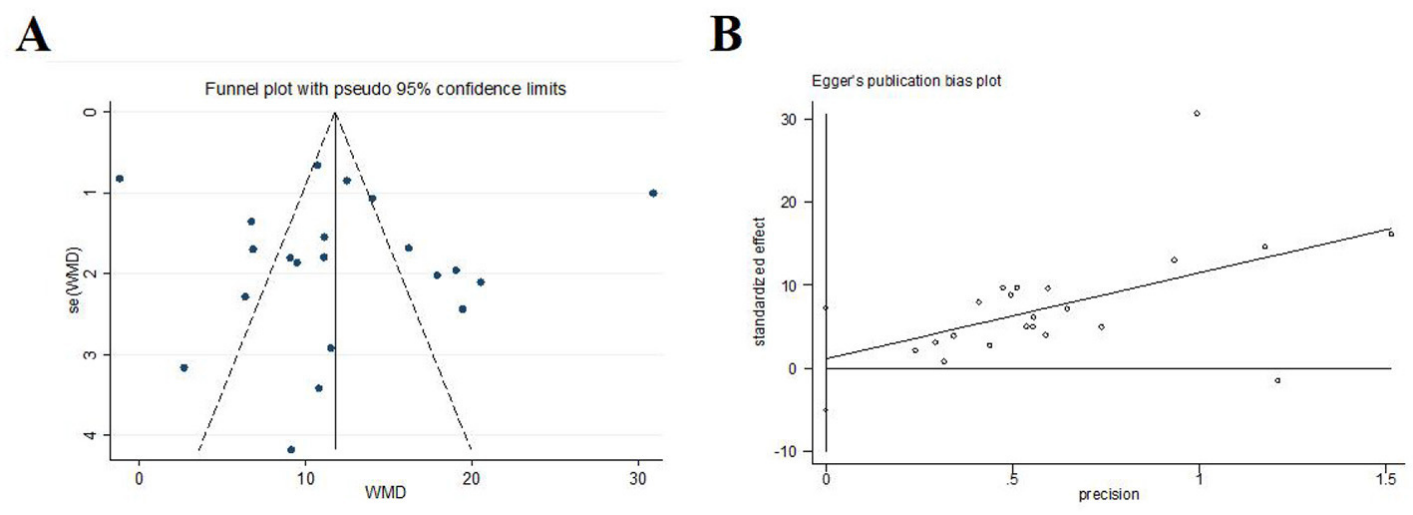
Publication bias in the daily living ability score. **(A)** Funnel plots. **(B)** Egger's test.

Twenty-one trials (*N* = 2,077) reported daily living ability score. The intervention group exhibited significantly higher daily living ability score compared to the control group [*MD* = 12.19, *95%CI* (8.50, 15.87), *I*^2^ = 97.2%, *P* < 0.001; Figure 9]. The plot on daily living ability score was visibly symmetric and Egger's test revealed no potential publication bias (*P* = 0.701; Figure 10).

#### Neurological deficit severity score

**Figure 11:**
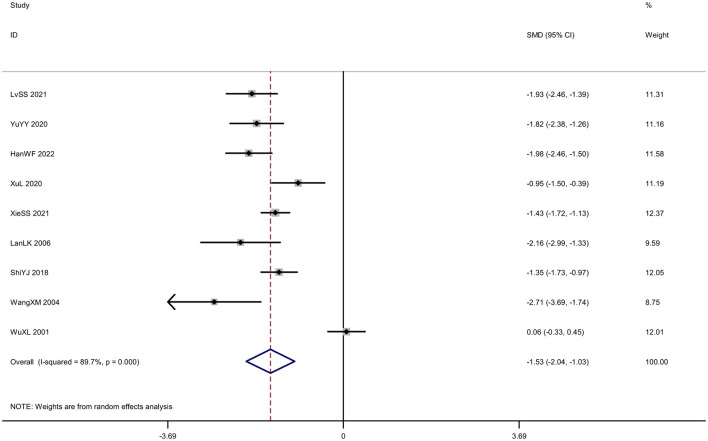
Forest plot of the neurological deficit severity score.

**Figure 12:**
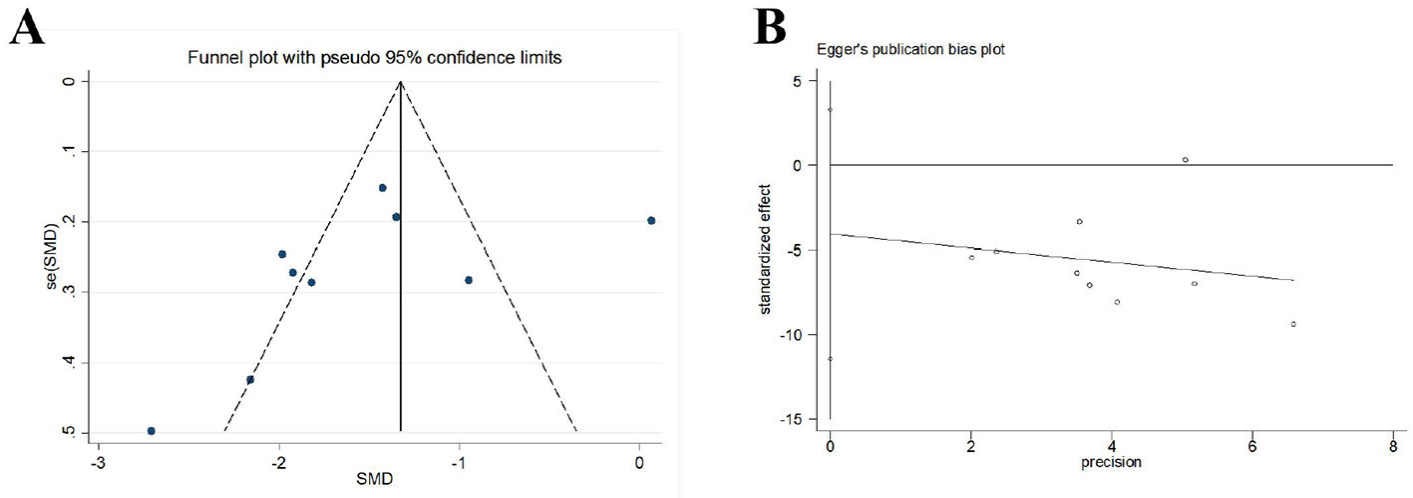
Publication bias in the neurological deficit severity score. **(A)** Funnel plots. **(B)** Egger's test.

A total of nine trials (*N* = 2,077) scored the severity of neurological deficits before and after treatment. Among them, five trials used the National Institutes of Health Stroke Scale (NIHSS) for scoring, and four trials used the Chinese stroke patient neurological deficit score, also known as the Modified Edinburgh-Scandinavian Stroke Scale (MESSS). Due to the use of different scales for scoring, the SMD was adopted as the indicator for the combined effect. The meta-analysis results showed that the experimental group was superior to the control group in reducing the neurological deficits of the subjects [*SMD* = –1.53, *95%CI* (–2.04, –1.03), *I*^2^ = 89.7%, *P* < 0.001; Figure 11). Funnel plots and Egger's test were done to evaluate publication bias, and results showed no significant publication bias (*P* = 0.233; Figure 12). Given the limited number of studies included for neurological deficit severity score, we also conducted a trim-and-fill analysis. No trimming performed and data unchanged indicated relatively stable results.

#### Safety assessment

**Figure 13:**
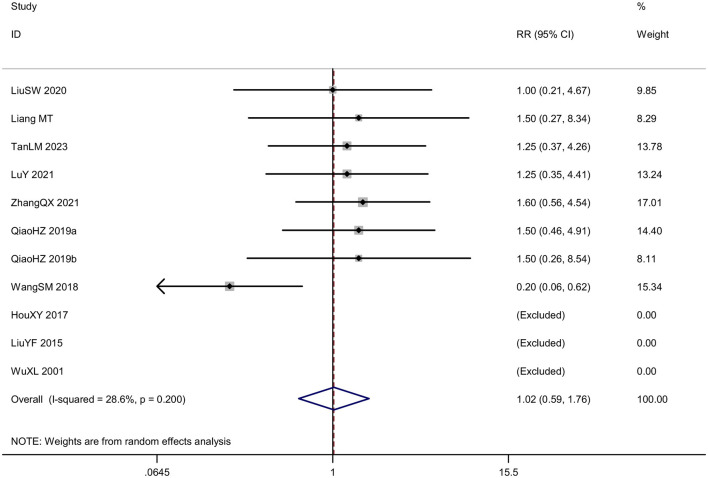
Forest plot of the safety assessment.

**Figure 14:**
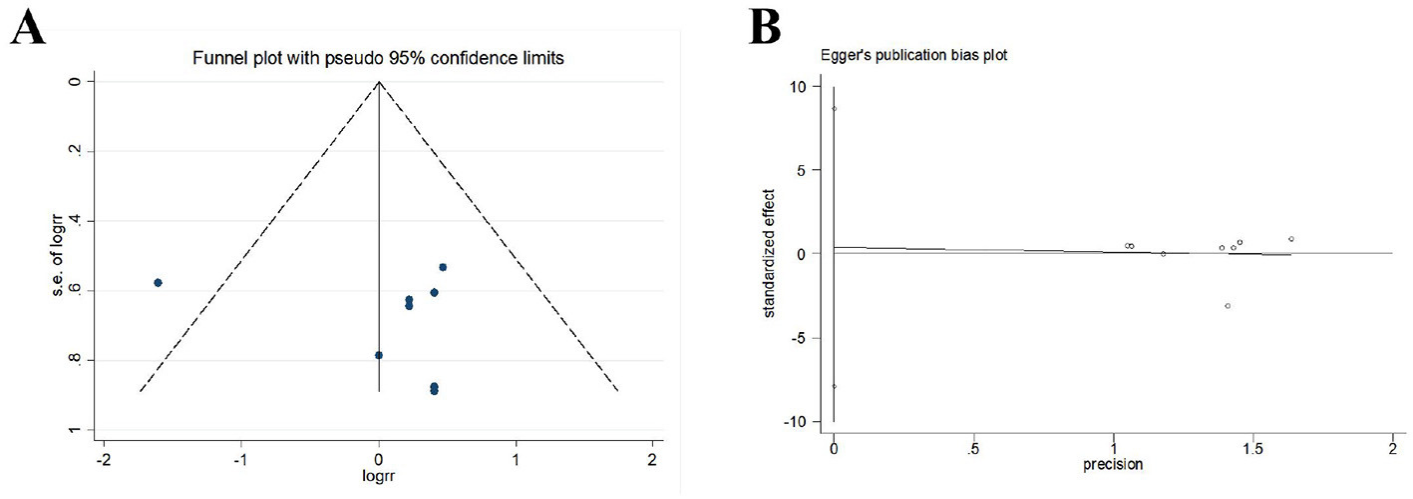
Publication bias in the safety assessment. **(A)** Funnel plots. **(B)** Egger's test.

Eleven RCTs reported the safety of acupuncture. Eight studies reported adverse events, including subcutaneous hemorrhage, dizziness, headache, fatigue, gastrointestinal discomfort, thirst, palpitation, cold sweat, etc. As an invasive treatment method, acupuncture can potentially puncture small blood vessels or veins within the subcutaneous tissue during the treatment process, leading to subcutaneous bleeding or hematoma. This is especially common for thin individuals with little subcutaneous fat, the elderly with increased vascular fragility, as well as areas with abundant capillaries such as the head and face, and areas close to veins. Therefore, doctors should consider the physical condition of the recipients to reduce the risk of subcutaneous bleeding or hematoma. Well, the subcutaneous hemorrhage usually absorbs on their own without requiring special treatment. However, not all subcutaneous bleeding or hematoma should be concealed, as blood-letting through acupuncture is also a traditional Chinese medical treatment method. As for this series of symptoms including dizziness, headache, fatigue, gastrointestinal discomfort, thirst, palpitation, cold sweat etc, they are a phenomenon known as acupuncture-related syncope. It may be related to the patient's weak constitution, mental tension, overexertion, hunger or improper positioning. Methods to handle syncope include immediately stopping the acupuncture, removing all the needles, supporting the patient to lie down flat for a while and providing warm water, after which recovery can occur. And three studies reported no adverse effects related to acupuncture. The incidence rate of adverse events in treatment group was 7.32% and that in control group was 8.04%. Analysis of data from adverse events showed low heterogeneity (*P* = 0.200, *I*^2^ = 28.6%), and the random-effects model showed no significant difference in the RR of acupuncture group compared with control group [RR = 1.02, 95% CI (0.59, 1.76); Figure 13]. A funnel plot was generated to visually assess potential publication bias and Egger test was conducted to assess publication bias. The results of the meta-analysis were not significantly affected by publication bias (*P* = 0.913; Figure 14).

#### Heterogeneity analysis

Given the pronounced heterogeneity, the subgroup analysis and meta-regression analysis were further conducted based on the interventions, comparisons, type of disease, course of disease and course of treatment in some outcomes including sensory disturbance score, visual analog scale and daily living ability score. The subgroup analysis and meta-regression analysis indicated no significant association between results and these potential influencing factors (Tables 3–6).

**Table 3:** Results of the subgroup meta analyses.

**Outcomes**	**Subgroup analyses**		***I* ^2^**	***P*-value**
Effective rate	Interventions	Warm acupuncture		
		Electroacupuncture	27.7%	0.002
		Acupuncture	9.4%	< 0.001
Sensory disturbance score	Interventions	Warm acupuncture		
		Electroacupuncture	94.2%	0.025
		Acupuncture	89.9%	< 0.001
	Comparisons	Rehabilitation	96.2%	< 0.001
		Basic treatment	90.3%	0.001
		Drug	25.0%	< 0.001
	Type of disease	Cerebrovascular disease	95.2%	< 0.001
		Ischemic cerebrovascular disease	20.8%	< 0.001
	Course of disease	Acute	94.8%	0.005
		Convalescence	92.8%	< 0.001
		Sequelae		
	Course of treatment	≤ 28 days	89.9%	< 0.001
		28–42 days	95.9%	0.029
		> 42 days	90.0%	< 0.001
Visual analog scale	Interventions	Electroacupuncture	75.4%	0.002
		Acupuncture	95.0%	< 0.001
	Comparisons	Rehabilitation	95.9%	0.020
		Basic treatment	98.0%	0.001
		Drug	61.2%	< 0.001
	Type of disease	Cerebrovascular disease	95.5%	< 0.001
		Ischemic cerebrovascular disease	74.8%	< 0.001
	Course of disease	Acute	95.3%	< 0.001
		Convalescence	95.2%	< 0.001
		Sequelae	17.8%	< 0.001
	Course of treatment	≤ 14 days		
		14–28 days	95.4%	< 0.001
		>28 days	85.4%	< 0.001
Daily living ability score	Interventions	Warm acupuncture		
		Electroacupuncture		
		Acupuncture	97.6%	< 0.001
		Fire needle		
	Comparisons	Rehabilitation	97.%	< 0.001
		Basic treatment	96.9%	0.019
		Drug	0.0%	< 0.001
Neurological deficit severity score	Interventions	Warm acupuncture		
		Acupuncture	90.4%	< 0.001
	Comparisons	Rehabilitation	68.6%	< 0.001
		Basic treatment	93.0%	< 0.001
	Type of disease	Cerebrovascular disease	92.3%	0.002
		Ischemic cerebrovascular disease	69.6%	< 0.001
	Course of disease	Acute	62.2%	< 0.001
		Convalescence	92.7%	< 0.001
		Sequelae		
	Course of treatment	≤ 20 days	96.6%	0.357
		20–40 days	62.7%	< 0.001
		> 40 days		

**Table 4:** Results of the meta-regression for the sensory disturbance score.

**Covariate**		**Coefficient (95% confidence interval)**	**P-value**
Interventions	Warm acupuncture	−0.51 (−0.69, 5.06)	0.844
	Acupuncture	−2.79 (−8.92, 3.34)	0.340
Comparisons	Rehabilitation	1.76 (−2.77, 6.28)	0.414
	Drug	0.61 (−3.34, 4.56)	0.741
Type of disease	Ischemic cerebrovascular disease	−1.17 (−4.92, 2.58)	0.513
Course of disease	Convalescence	−0.02 (−3.83, 3.78)	0.990
	Sequelae	−1.71 (−8.75, 5.34)	0.607
Course of treatment	≤ 28 days	−1.96 (−5.48, 1.55)	0.248
	28–42 days	1.11 (−3.77, 5.98)	0.629

**Table 5:** Results of the meta-regression for the visual analog scale.

**Covariate**		**Coefficient (95% confidence interval)**	**P-value**
Interventions	Acupuncture	0.06 (−1.53, 1.66)	0.934
Comparisons	Basic treatment	−1.21 (−2.54, 0.12)	0.073
	Rehabilitation	0.02 (−1.15, 1.19)	0.973
Type of disease	Ischemic cerebrovascular disease	−0.39 (−1.38, 0.60)	0.422
Course of disease	Acute	0.05 (−1.97, 2.07)	0.956
	Convalescence	0.08 (−1.61, 1.77)	0.924
Course of treatment	≤ 14 days	1.16 (−1.26, 3.58)	0.328
	14–28 days	−0.37 (−1.51, 0.77)	0.507

**Table 6:** Results of the meta-regression for the daily living ability score.

**Covariate**		**Coefficient (95% confidence interval)**	**P-value**
Interventions	Warm acupuncture	6.07 (−9.85, 21.99)	0.432
	Acupuncture	3.98 (−18.80, 26.76)	0.717
	Fire needle	−0.45 (−22.59, 21.69)	0.966
Comparisons	Rehabilitation	−0.94 (−11.87, 9.99)	0.859
	Drug	3.30 (−6.68, 13.28)	0.496
Type of disease	Ischemic cerebrovascular disease	0.67 (−8.10, 9.43)	0.875
Course of disease	Acute	9.48 (−3.59, 22.55)	0.145
	Convalescence	5.69 (−5.80, 17.17)	0.312
Course of treatment	≤ 20 days	−7.42 (−18.07, 3.23)	0.160
	20–40 days	−5.93 (−15.25, 3.40)	0.198

#### Sensitivity analysis

**Figure 15:**
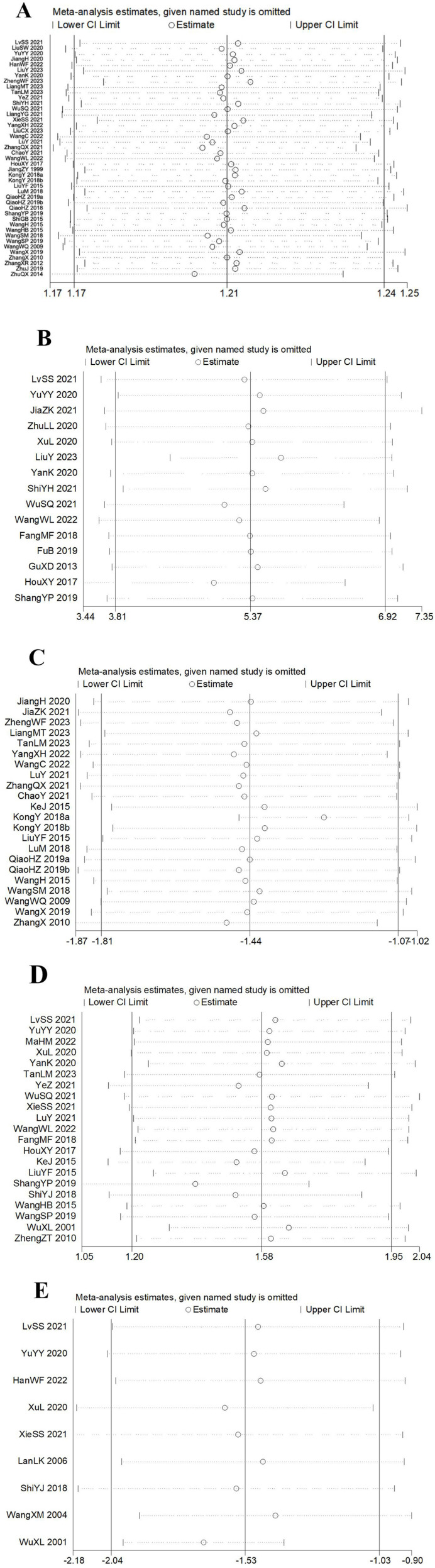
Sensitivity analysis. **(A)** Effective rate. **(B)** Sensory disturbance score. **(C)** Visual analog scale. **(D)** Daily living ability score. **(E)** Neurological deficit severity score.

Further sensitivity analysis was performed to assess the stability of the outcomes of meta-analysis. And the results intuitively showed the robust of the outcomes (Figure 15).

### Certainty of evidence

The results of the GRADE analysis showed that sensory disturbance score and VAS were ranked as high-quality, effective rate and daily living ability score were ranked as moderate-quality and other results were ranked as low-quality (Table 7).

**Table 7:** GRADE summary of outcomes.

**Certainty assessment**							**No of patients**		**Effect (95% CI)**		**Certainty**	**Importance**
**No**.	**Study design**	**Risk of bias**	**Inconsistency**	**Indirectness**	**Imprecision**	**Other considerations**	**T**	**C**	**Relative**	**Absolute**		
**Effective rate**												
43	RCT	Not serious	Serious^a^	Not serious	Not serious	Not serious	1,744/1,900 (91.8%)	1,412/1,898 (74.4%)	RR 1.24 (1.20 to 1.27)	–	⊕⊕⊕○ Moderate	Important
**Sensory disturbance score**												
15	RCT	Not serious	Not serious	Not serious	Not serious	Not serious	704	703	–	MD 5.37 higher (3.81 higher to 6.92 higher)	⊕⊕⊕⊕ High	Important
**Visual analog scale**												
22	RCT	Not serious	Not serious	Not serious	Not serious	Not serious	825	826	–	MD 1.44 lower (1.81 lower to 1.07 lower)	⊕⊕⊕⊕ High	Important
**Daily living ability score**												
21	RCT	Not serious	Serious^a^	Not serious	Not serious	Not serious	1,041	1,036	–	MD 12.19 higher (8.5 higher to 15.87 higher)	⊕⊕⊕○ Moderate	Important
**Neurological deficit severity score**												
9	RCT	Not serious	Serious^a^	Not serious	Serious^b^	Not serious	415	411	–	SMD 1.53 lower (2.04 lower to 1.03 lower)	⊕⊕○○ Low	Important
**Safety assessment**												
11	RCT	Not serious	Serious^a^	Not serious	Serious^b^	Not serious	36/451 (8.0%)	36/449 (8.0%)	RR 0.92 (0.60 to 1.41)	–	⊕⊕○○ Low	Important

CI, confidence interval; MD, mean difference; SMD, standardized mean difference.

^a^The result of one RCT is inconsistent with the overall combined effect size.

^b^The sample size is <1,000 cases.

## Discussion

### Summary of main results

This study included a total of 57 randomized controlled trials (RCTs), with 55 in Chinese and two in English. The RoB tool evaluation indicated that the high-risk items in the included literature were mainly due to the lack of specific descriptions of the random sequence concealment method, and the failure to use blinding for patients, intervention implementers, data analysts, data collectors, and outcome evaluators, with only a few documents reporting dropout situations. The GRADE evaluation results showed that the evidence quality for the severity of neurological deficit scores and adverse event occurrence was low.

The results revealed the following.

Compared with control group, acupuncture is superior in improving efficiency, alleviating sensory disorders, reducing pain, enhancing daily living abilities, and relieving neurological deficits for sensory disorders after stroke.
There was no statistically significant difference in the incidence of adverse events between the treatment group and the control group.

### In-depth analysis of meta-analysis results

Funnel plots were drawn for outcome indicators such as efficiency, sensory disorder scores, visual analog scale scores, and daily living abilities, and the symmetry of the funnel plots was tested using the Egger method. The results showed that the funnel plots were basically symmetrical, indicating that the probability of publication bias in the included literature was small.

For outcome indicators with large heterogeneity, including sensory disorder scores, visual analog scale scores, daily living abilities, and the severity of neurological deficit scores, subgroup analysis and meta-regression were conducted to explore the sources of heterogeneity according to different experimental group interventions, control group interventions, disease types, course of disease, and treatment courses. The results showed that these factors were not the reasons for the high heterogeneity. Except for the post-sequelae phase subgroup in the daily living abilities outcome indicator and the treatment course ≤ 20 days subgroup in the neurological deficit severity score outcome indicator, the results of the other outcome indicators' subgroup analyses were statistically significant (*P* < 0.05), which can indirectly indicate that the meta-analysis results of the above outcome indicators have good robustness. The reasons for the lack of statistical significance in the subgroup results may be the following two points: (1) The number of included RCTs and sample sizes is small, with only two RCTs included in each of the two subgroups, and the sample size of both groups is <200 cases, which led to the appearance of false-negative results; (2) The therapeutic effect of acupuncture may be related to the intervention time and the treatment course. Ruihao's study (73) showed that in the real-world study of comprehensive acupuncture therapy intervention for ischemic stroke, the earlier the intervention of comprehensive acupuncture therapy, the more treatment times, the better the improvement effect on patients' neurological deficits.

To test the robustness of the meta-analysis results, sensitivity analysis was performed by comparing the meta-analysis results under different effect models and the meta-analysis results after removing a single study, and the results showed that the meta-analysis results of each outcome indicator had good robustness.

### Overall completeness and applicability of evidence

In this systematic review, all the studies included were undertaken solely in China, with a significant portion of them being published in the Chinese language. However, we acknowledge the possibility that we may have overlooked some pertinent trials, particularly those that were not published in journals indexed by the electronic databases we utilized for our search.

### Characteristics of participants

The studies included in this review were primarily conducted in China, limiting the review's generalizability across racial groups. The responsible vessel for cerebrovascular disease, variations in stroke types and severities could potentially modify the effectiveness of acupuncture on stroke patients. Unfortunately, given the constraints, including the scarcity of studies focusing on specific cerebral blood vessel, inconsistent reporting of stroke severity across trials, and ambiguity in defining the timing of treatment initiation post-stroke, it was not feasible to conduct predetermined subgroup analyses comparing patients with varying stroke severities or those who began treatment at different time points after their stroke. Consequently, the findings may not fully capture the nuances of acupuncture's impact across diverse stroke populations and treatment timelines.

### The highlights of this study

Currently, no meta-analysis of acupuncture treatment for sensory disorders after stroke has been found to be publicly published. This study is based on clinical needs, formulates rigorous retrieval strategies and inclusion and exclusion criteria, and conducts a comprehensive search in 14 commonly used domestic and international databases and clinical trial registration websites, strictly following the principle of double-blind independent work in literature screening, data extraction, and quality evaluation. Internationally recognized tools, the Jadad scale and the modified RoB tool, are used to comprehensively and objectively evaluate the quality of the included RCTs.

### Limitations

This systematic review and meta-analysis also had several limitations. First, this study only included articles conducted in China, which might limit the reliability and global applicability of our findings. When discussing the applicability of acupuncture among non-Chinese populations, the cultural factors or clinical practice variations cannot be overlooked. Acupuncture originated from Traditional Chinese Medicine (TCM), within the framework of the Yin-Yang and Five Elements theory. It may be challenging for non-Chinese populations to comprehend the theories of TCM. Additionally, shortages of skilled personnel and a lack of foundational research pose challenges to the internationalization of acupuncture. On the other hand, eastern and western cultures have distinct characteristics. Asians, especially Chinese people under the traditional Chinese medicine framework, may experience a stronger placebo effect. Experimental designs to exclude acupuncture placebo effects are constantly improving, with methods like fake needles and sham acupoints seeking better forms to minimize placebo effects. Based on studies conducted solely with Chinese participants, we may overestimate the true efficacy and safety of acupuncture. Second, only a small portion of the included literature reported adverse events and the reports lacked detail. The lack of reporting on adverse events may increase patient risks, lead to an underestimation of treatment-related injuries and thereby affect clinical decision-making. Third, the methodological quality of the evidence in this study was not high. Only one included trial reported the implementation of allocation concealment. Moreover, due to the particularity of acupuncture, it is difficult to implement a blind design. The lack of blinding and randomization concealment may lead to selective reporting bias, exaggerating treatment effects or underestimating adverse reactions. No studies included a placebo-controlled group, which significantly impacted the assessment of efficacy and posed a considerable limitation when drawing conclusions about the overall effectiveness of acupuncture. Fourth, there was high heterogeneity in the data, which may limit the generalizability of the findings.

### Inspiration and suggestions for future research

First, it is recommended to enhance international communication and develop a unified acupuncture protocol. We suggest that future clinical trials conducting multicenter trials involving diverse populations to improve external validity. Second, we suggest that future clinical trials could systematically collect and report safety outcomes. Third, we suggest that future clinical trials can refer to the corresponding methodological quality evaluation tools for design, and the design in aspects such as random sequence generation, concealment, and blinding should be more scientifically rigorous. Furthermore, the formulation of inclusion criteria should refer to the internationally recognized diagnostic criteria. The baseline data of the included samples should be basically consistent, and the gender, age, course of disease, and severity of the disease of the included patients should be recorded in detail, and the intervention measures and outcome measurement information should be reported as detailed as possible in the registered trial protocol. It is meaningful for future studies to explore innovative methods to simulate the sensation of acupuncture and ensure the effectiveness of blinding. In conclusion, we propose that future research establish a standardized acupuncture protocol to minimize heterogeneity. We hope a scientific and rigorous research paradigm will emerge, promoting practical acupuncture applications both in and outside China, revealing its exact meridian mechanisms.

## Conclusion

The best available clinical research evidence indicates that acupuncture for sensory disorders after stroke has certain advantages over the control group in improving efficiency, alleviating sensory disorders, reducing pain, enhancing daily living abilities, and relieving neurological deficits. Additionally, the adverse reactions in acupuncture group were acceptable and the incidence of adverse events is lower than in the control group. However, due to the low quality of the evidence, clinical decision-making should still be made with caution. More high-quality clinical trials are still needed in the future to further verify the clinical efficacy and safety. In order to enhance the universality of acupuncture and promote the dissemination of traditional Chinese medicine worldwide, we look forward to the initiation of more international clinical trials regarding acupuncture treatment for stroke.

## Data Availability

The original contributions presented in the study are included in the article/Supplementary material, further inquiries can be directed to the corresponding authors.
