# Supplementary material for: Acupuncture for somatosensory deficits after stroke: a systematic review and meta-analysis
Source: Front Med (Lausanne). 2025 Feb 7;12:1504215. doi: 10.3389/fmed.2025.1504215 (PMC11841453; doi:10.3389/fmed.2025.1504215)
Supplement: Supplementary file 1 [file Table_1.docx]

| Study ID | Treatment group | Control group |
| --- | --- | --- |
|  | Acupoint | Intervention |
| LvSS 2021 | *neiguan* (PC6), *baihui* (DU20), *quchi* (LI11), *fenglong* (ST40), *hegu* (LI4), *yanglingquan* (GB34), *sanyinjiao* (SP6), *Ah Shi points* | rehabilitation |
| LiuSW 2020 | *baihui* (DU20), *fengchi* (GB20), *hegu* (LI4), *zusanli* (ST36), *sanyinjiao* (SP6) | basic treatment |
| YuYY 2020 | *jianyu* (LI15), *quchi* (LI11), *shousanli* (LI10), *biguan* (ST31), *fengshi* (GB31), *futu* (ST32), *zusanli* (ST36), *yanglingquan* (GB34), *fenglong* (ST40), *Ah Shi points* | basic treatment |
| JiangH 2020 | *shuigou* (GV26), *neiguan* (PC6), *sanyinjiao* (SP6), *jiquan* (HT1), *weizhong* (BL40), *fengchi* (GB20), *wangu* (GB12), *tianzhu* (BL10), *zusanli* (ST36), *yanglingquan* (GB34), *fenglong* (ST40) | basic treatment |
| MaHM 2022 | *jianyu* (LI15), *binao* (LI14), *quchi* (LI11), *wenliu* (LI7), *hegu* (LI4), *biguan* (ST31), *futu* (ST32), *liangqiu* (ST34), *zusanli* (ST36), *shangjuxu* (ST37), *jiexi* (ST41) | rehabilitation |
| JiaZK 2021 | *baihui* (DU20), *naohu* (GV17), *dazhui* (GV14), *zhiyang* (GV9), *jinsuo* (GV8), *xuanshu* (GV5), *yaoyangguan* (GV3), *changqiang* (GV1) | Pregabalin |
| HanWF 2022 | / | basic treatment |
| ZhuLL 2020 | *shenting* (GV24), *baihui* (DU20), *fengfu* (GV16), *yamen* (GV15), *dazhui* (GV14), *zhiyang* (GV9), *yaoyangguan* (GV3), *mingmen* (GV4) | rehabilitation |
| XuL 2020 | Jiao's Scalp Acupuncture | rehabilitation |
| LiuY 2023 | Wrist-Ankle Acupuncture | rehabilitation |
| YanK 2020 | Wrist-Ankle Acupuncture | Methylcobalamin |
| ZhengWF 2023 | *shuigou* (GV26), *neiguan* (PC6), *sanyinjiao* (SP6), *fengchi* (GB20), *wangu* (GB12), *tianzhu* (BL10), *jiquan* (HT1), *weizhong* (BL40) | Pregabalin |
| LiangMT 2023 | *shuigou* (GV26), *neiguan* (PC6), *sanyinjiao* (SP6), *jiquan* (HT1), *weizhong* (BL40), *chize* (LU5), *fengchi* (GB20), *wangu* (GB12), *tianzhu* (BL10) | Pregabalin |
| TanLM 2023 | *sanyinjiao* (SP6), *shuigou* (GV26), *neiguan* (PC6), *tianzhu* (BL10), *jiquan* (HT1), *weizhong* (BL40), *fengchi* (GB20), *wangu* (GB12) | Amitriptyline |
| YeZ 2021 | *shuigou* (GV26), *neiguan* (PC6) | rehabilitation |
| ShiYH 2021 | Eye Acupuncture | basic treatment |
| WuSQ 2021 | *baihui* (DU20), *yintang* (GV29), *fengchi* (GB20), *dicang* (ST4), *taiyang*, *quchi* (LI11), *hegu* (LI4), *jianliao* (SJ14), *binao* (LI14), *naoshu* (SJ10), *neiguan* (PC6), *waiguan* (SJ5), *shousanli* (LI10), *zusanli* (ST36), *sanyinjiao* (SP6), *fenglong* (ST40), *taichong* (LR3), *xuehai* (SP10), *yanglingquan* (GB34) | rehabilitation |
| LiangYG 2021 | *baihui* (DU20), *fengfu* (GV16), *yamen* (GV15), *shenting* (GV24), *shuigou* (GV26), *dazhui* (GV14), *zhiyang* (GV9), *yaoyangguan* (GV3), *changqiang* (GV1) | basic treatment |
| XieSS 2021 | *jiquan* (HT1), *fengchi* (GB20), *shuigou* (GV26), *neiguan* (PC6), *taixi* (KI3) | basic treatment |
| YangXH 2022 | Jiao's Scalp Acupuncture, *shuigou* (GV26) | Carbamazepine |
| LiuCX 2023 | *pishu* (BL20), *xinshu* (BL15), *feishu* (BL13), *shenshu* (BL23), *ganshu* (BL18) | basic treatment |
| WangC 2022 | *shuigou* (GV26), *shenting* (GV24), *baihui* (DU20), *yongquan* (KI1), *laogong* (PC8) | Pregabalin |
| LuY 2021 | *shuigou* (GV26), *neiguan* (PC6), *sanyinjiao* (SP6) | Amitriptyline |
| ZhangQX 2021 | *ximen* (PC6), *yinxi* (HT7), *xuehai* (SP10), *zhaohai* (KI6) | Carbamazepine |
| ChaoY 2021 | Wrist-Ankle Acupuncture, Jiaji points | Carbamazepine |
| WangWL 2022 | *jianyu* (LI15), *binao* (LI14), *quchi* (LI11), *waiguan* (SJ5), *hegu* (LI4), fengshi (GB31), *weizhong* (BL40), *zusanli* (ST36), *yanglingquan* (GB34), *sanyinjiao* (SP6) | basic treatment |
| FangMF 2018 | Wrist-Ankle Acupuncture | rehabilitation |
| FuB 2019 | Wrist-Ankle Acupuncture | Methylcobalamin |
| GuXD 2013 | Scalp Acupuncture | rehabilitation |
| HouXY 2017 | Scalp Acupuncture, *fengchi* (GB20), *waiguan* (SJ5), *zusanli* (ST36), *yongquan* (KI1), *quchi* (LI11) | basic treatment |
| JiangZY 1999 | Jiaji points | Carbamazepine |
| KeJ 2015 | Balance Acupuncture | rehabilitation |
| KongY 2018 | Scalp Acupuncture | Pregabalin |
| LanLK 2006 | *neiguan* (PC6), *shuigou* (GV26), *sanyinjiao* (SP6) | basic treatment |
| LiuYF 2015 | *neixiyan*, *dubi* (ST35), *xuehai* (SP10), *liangqiu* (ST34) | basic treatment |
| LuM 2018 | *shuigou* (GV26), *neiguan* (PC6), *sanyinjiao* (SP6), *jiquan* (HT1), *weizhong* (BL40), *fengchi* (GB20), *wangu* (GB12), *tianzhu* (BL10) | Carbamazepine |
| QiaoHZ 2019a | Scalp Acupuncture | Pregabalin, Gabapentin |
| QiaoHZ 2019b | *yuyao*, *yangbai* (GB14), *xiaguan* (ST7), *jiache* (ST6), *chengjiang* (ST24), *dicang* (ST4), *sibai* (ST2), *shuigou* (GV26), *yingxiang* (LI20), *baihui* (GV20), *qubin* (GB7), *taiyang*, *neiguan* (PC6), waiguan (SJ5), danzhong (CV17), zhongting (CV18), hegu (LI4), *zusanli* (ST36) | basic treatment |
| QiaoHZ 2018 | *baihui* (DU20), *taiyang* | basic treatment |
| ShangYP 2019 | Scalp Acupuncture | rehabilitation |
| ShiGB 2015 | Dong's Acupuncture Points | basic treatment |
| ShiYJ 2018 | *zusanli* (ST36), *yongquan* (KI1), *fenglong* (ST40), *hegu* (LI4), *yanglingquan* (GB34), *weizhong* (BL40), *shuigou* (GV26), *neiguan* (PC6), *quchi* (LI11), *baihui* (DU20), *sanyinjiao* (SP6), *chize* (LU5) | rehabilitation |
| WangH 2015 | *baihui* (DU20), *taiyang* | Amitriptyline |
| WangHB 2015 | *baihui* (DU20), *yintang* (GV29), *yingxiang* (LI20), *taiyang*, *dicang* (ST4), *shuigou* (GV26), *fengchi* (GB20), *jianliao* (SJ14), *quchi* (LI11), *shousanli* (LI10), *hegu* (LI4), *neiguan* (PC6), *waiguan* (SJ5), *huantiao* (GB29), *zusanli* (ST36), *sanyinjiao* (SP6), *yanglingquan* (GB34), *xuehai* (SP10), *yinlingquan* (SP9) | rehabilitation |
| WangSM 2018 | Scalp Acupuncture | Pregabalin |
| WangSP 2019 | *baihui* (GV20), *shenting* (GV24), *binao* (LI14), *houxi* (SI3), *baxie*, *jianyu* (LI15), *quchi* (LI11), *chize* (LU5), *neiguan* (PC6), *shousanli* (LI10), *zusanli* (ST36), *xuehai* (SP10), *fengchi* (GB20), *hegu* (LI4), *yangxi* (LI5), *jianliao* (SJ14), *jianzhen* (SI9), *taichong* (LR3), *Ah Shi points* | rehabilitation |
| WangWQ 2009 | / | Carbamazepine |
| WangX 2019 | Scalp Acupuncture | basic treatment |
| WangXM 2004 | *neiguan* (PC6), *shuigou* (GV26), *sanyinjiao* (SP6) | basic treatment |
| WuXL 2001 | *baihui* (DU20), *sanyinjiao* (SP6), *taixi* (KI3), *xuehai* (SP10), *zusanli* (ST36), *fenglong* (ST40) | basic treatment |
| XingYL 2007a | Scalp Acupuncture | rehabilitation |
| XingYL 2007b | Scalp Acupuncture | basic treatment |
| ZhangX 2010 | *ximen* (PC6), *yinxi* (HT7), *xuehai* (SP10), *zhaohai* (KI6) | Carbamazepine |
| ZhangXR 2012 | *ximen* (PC6), *xuehai* (SP10), Jiaji points | Methylcobalamin, Carbamazepine |
| ZhangYE 2015 | Scalp Acupuncture | basic treatment |
| ZhengZT 2010 | *jianyu* (LI15), *quchi* (LI11), *shousanli* (LI10), *waiguan* (SJ5), *hegu* (LI4), *sanjian* (LI3), *huantiao* (GB29), *zusanli* (ST36), *sanyinjiao* (SP6), *fengshi* (GB31), *taichong* (LR3), *jiexi* (ST41) | rehabilitation |
| ZhuJ 2019 | / | basic treatment |
| ZhuQX 2014 | / | Methylcobalamin |
