# Supplementary material for: Acupuncture for somatosensory deficits after stroke: a systematic review and meta-analysis
Source: Front Med (Lausanne). 2025 Feb 7;12:1504215. doi: 10.3389/fmed.2025.1504215 (PMC11841453; doi:10.3389/fmed.2025.1504215)
Supplement: Supplementary file 2 [file Table_2.docx]

**Appendix 2.** Results of the subgroup analyses.

**1.Effective rate**

(1) Results of the subgroup analysis based on interventions

**2.Sensory disturbance score**

(1) Results of the subgroup analysis based on interventions

(2) Results of the subgroup analysis based on comparisions

(3) Results of the subgroup analysis based on type of disease

(4) Results of the subgroup analysis based on course of disease

(5) Results of the subgroup analysis based on course of treatment

a (≤28 d) vs b (28~42d) vs c (＞42d)

**3.Visual analog scale**

(1) Results of the subgroup analysis based on interventions

(2) Results of the subgroup analysis based on comparisions

(3) Results of the subgroup analysis based on type of disease

(4) Results of the subgroup analysis based on course of disease

(5) Results of the subgroup analysis based on course of treatment

a (≤14d) vs b (14~28d) vs c (＞28d)

**4.Daily living ability score**

(1) Results of the subgroup analysis based on interventions

(2) Results of the subgroup analysis based on comparisions

(3) Results of the subgroup analysis based on type of disease

(4) Results of the subgroup analysis based on course of disease

(5) Results of the subgroup analysis based on course of treatment

a (≤20d) vs b (20~40d) vs c (＞40d)

**5.Neurological deficit severity score**

(1) Results of the subgroup analysis based on interventions

(2) Results of the subgroup analysis based on comparisions

(3) Results of the subgroup analysis based on type of disease

(4) Results of the subgroup analysis based on course of disease

(5) Results of the subgroup analysis based on course of treatment

a (≤20d) vs b (20~40d) vs c (＞40d)
